# Magnetically guided surgery after primary systemic therapy for breast cancer: implications for enhanced axillary mapping

**DOI:** 10.1093/bjs/znae008

**Published:** 2024-02-07

**Authors:** Eirini Pantiora, Staffan Eriksson, Fredrik Wärnberg, Andreas Karakatsanis

**Affiliations:** Department of Surgical Sciences, Uppsala University, Uppsala, Sweden; Section for Breast Surgery, Department of Surgery, Uppsala University Hospital, Uppsala, Sweden; Centre for Clinical Research, Department of Surgical Sciences, Uppsala University, Västerås, Sweden; Section for Breast Surgery, Department of Surgery, Västmanlands County Hospital, Västerås, Sweden; Sahlgrenska Centre for Cancer Research, Department of Surgery, Institute of Clinical Sciences, Sahlgrenska Academy, University of Gothenburg, Gothenburg, Sweden; Department of Surgery, Sahlgrenska University Hospital, Gothenburg, Sweden; Department of Surgical Sciences, Uppsala University, Uppsala, Sweden; Section for Breast Surgery, Department of Surgery, Uppsala University Hospital, Uppsala, Sweden

## Abstract

**Background:**

Superparamagnetic iron nanoparticles perform comparably to radioisotope ± blue dye for sentinel lymph node detection in breast cancer, even when injected up to 8 weeks before surgery. Using superparamagnetic iron nanoparticles for sentinel lymph node detection after primary systemic therapy, and the maximum time frame of superparamagnetic iron nanoparticle administration have not been investigated.

**Methods:**

This cohort study included cN0/1-to-ycN0 patients undergoing sentinel lymph node detection or targeted axillary dissection. All patients received superparamagnetic iron nanoparticles either before primary systemic therapy or before surgery, and radioisotope on the day of surgery.

**Results:**

For 113 patients analysed, superparamagnetic iron nanoparticles were injected a median of 3 (range 0–248) days before surgery, with a 97.4% detection rate compared with 91.2% for radioisotope (*P* = 0.057). Concordance for radioisotope was 97.1% and this was not affected by timing of superparamagnetic iron nanoparticle injection (Kendall’s tau 0.027; *P* = 0.746). The median sentinel lymph node yield was 3 (interquartile range (i.q.r.) 2–3) for superparamagnetic iron nanoparticles and 2 (i.q.r. 2–3) for radioisotope (*P* < 0.001). In targeted axillary dissection, detection was 100% for superparamagnetic iron nanoparticles and 81.8% for radioisotope (*P* = 0.124). The index node was magnetic in 93.9% and radioactive in 66.7% (*P* = 0.007), an outcome that was not affected by any factors. For patients with metastases, superparamagnetic iron nanoparticle detection was 100% and radioisotope-based detection was 84.2% (*P* = 0.083), with superparamagnetic iron nanoparticles detecting more metastatic sentinel lymph nodes (median of 1 (i.q.r. 1–2) for superparamagnetic iron nanoparticles compared with a median of 1 (i.q.r. 0–1) for radioisotope; *P* = 0.005).

**Conclusion:**

Injection before primary systemic therapy is feasible and does not affect concordance with radioisotope. Superparamagnetic iron nanoparticles perform comparably to radioisotope, but detect more sentinel lymph nodes and have a higher rate of detection of metastatic sentinel lymph nodes.

## Teaser

In this study, patients with breast cancer who received oncological treatment before surgery underwent axillary surgery by means of a superparamagnetic iron nanoparticle tracer, which was injected at any time point, ranging from the day before surgery to before the start of oncological treatment. All patients received a radioisotope tracer, the standard, as a comparator. The superparamagnetic iron nanoparticle detection rate and accuracy for sentinel lymph node detection were not affected by the time of injection (before or after preoperative oncological therapy) and superparamagnetic iron nanoparticles detected more lymph nodes, especially for patients with nodal metastases.

## Graphical Abstract

**Graphical abstract:**
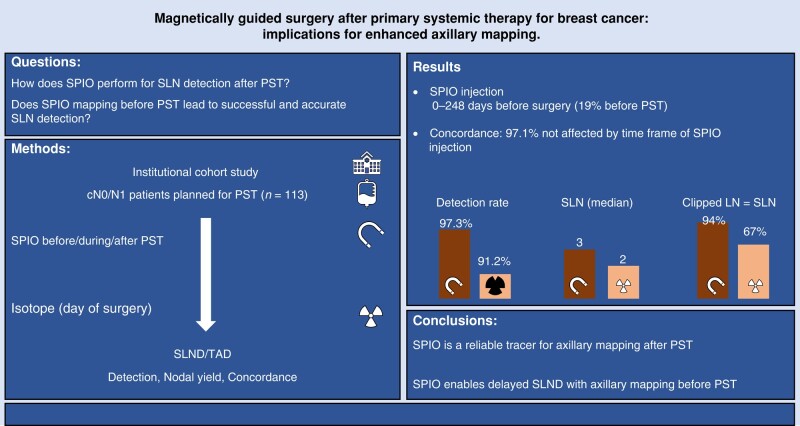

## Introduction

Superparamagnetic iron nanoparticles (SPIO) have shown comparable performance to radioisotope ± blue dye for sentinel lymph node (SLN) detection (SLND) in breast cancer, with the convenience of easier accessibility, disposal, and administration days before surgery^1^. Moreover, SPIO provide the possibility for delayed SLND, as demonstrated in the SentiNot study. In that study, SLND using SPIO was still feasible weeks after primary breast surgery for ductal carcinoma *in situ*, in the cases where specimen pathology demonstrated invasive cancer^2,3^. However, the role of SPIO for SLND after primary systemic therapy (PST) has not been extensively investigated.

Current evidence suggests that radioisotope-based SLND is the accepted standard after PST. Radioisotope-based dual mapping is specifically recommended for cN+-to-ycN0 patients, when SLN biopsy or targeted axillary dissection (TAD) is performed, as the number of SLN retrieved has been inversely linked to the false negative rate^4–8^. Apart from the logistic benefits of SPIO, with administration before PST, an additional aspect of interest is the ability to map the axilla before the fibrotic changes and lymphatic remodelling induced by chemotherapy occur, a concern mainly in patients who are initially cN+^9^. This mandates investigating that SPIO remain detectable after a prolonged interval of administration and that they do not migrate to higher nodal echelons. While preliminary data suggest feasibility^10^, the aim of this study was to investigate the width of time frame of SPIO administration for patients undergoing PST and the concordance of SPIO and radioisotope-based detection.

## Methods

### Inclusion process

This study considered patients with non-metastatic, non-inflammatory breast cancer and cN0/cN1 axillae, intended for PST (chemotherapy, targeted therapy, or endocrine therapy) with curative intent, recruited at Uppsala University Hospital between January 2020 and October 2022. Tumour progression during PST and surgery before the completion of PST for any reason (for example PST adverse effects and patient preference) were exclusion criteria. For cN+-to-ycN0 patients, a decision regarding TAD was taken after discussion at the multidisciplinary meeting, followed by patient consent, as, during the enrolment interval, TAD was not yet included in the Swedish National Guidelines. Patients who opted for upfront axillary lymph node dissection were also excluded from this study. The final study cohort consisted of ycN0 patients, scheduled for either SLND alone or TAD.

### Procedures

Initial diagnostic workup consisted of mammogram, breast/axillary ultrasonography, and core biopsy. In cases with a single palpable axillary lymph node or up to three suspicious axillary lymph nodes on ultrasonography in the absence of palpable lymphadenopathy, the most prominent lymph node was sampled by either fine-needle aspiration or core biopsy, according to radiologist preference. The lymph nodes were clipped during the same session at the discretion of the radiologist with a conventional marker. If fine-needle aspiration or core biopsy was negative, but clinical suspicion was high (BI-RADS 5, corresponding to a lymph node with metastatic features), removal was *a priori* intended. For patients with biopsy-proven metastatic lymph nodes, but without bulky axillary lymphadenopathy, a discussion regarding the possibility of de-escalation in the case of response to treatment took place in the multidisciplinary meeting. Initially, conventional clips were placed and replaced with paramagnetic clips (Magseed^®^; Endomag, Cambridge, UK) before surgery, but, later in this study, paramagnetic clips were used directly for biopsy-proven metastatic lymph nodes. This practice extended to patients in need of MRI monitoring, with the exception of axillary tail tumours^11^. For patients requiring MRI monitoring, SPIO (Magtrace^®^; Endomag; 1 ml) were administered peritumorally or in the clip of the residual tumour after PST completion and before surgery, either during the preoperative surgical consultation or during lesion localization by the radiologist. If MRI monitoring was not necessary, SPIO were injected peritumorally before or after PST initiation. All patients received radioisotope on the day of surgery (40 mBq) or the day before (60 mBq), divided into two doses (periareolar and at the tumour bed), according to local routines. Axillary surgery (SLND or TAD) was performed under magnetic probe guidance (Sentimag^®^; Endomag) and the resected lymph nodes were controlled for magnetic and then radioactive signal *ex vivo*. Upon completion of the procedure with the magnetic probe, the axilla was controlled with the radioisotope probe and any additional lymph nodes with a radioactive, but not magnetic, signal were removed. Clinically enlarged and suspicious lymph nodes were also removed in line with preoperative patient consent. Accordingly, in TAD cases, if less than two SLN were retrieved and the index node was detected, enlarged lymph nodes detected during surgery or axillary lymph node dissection were removed, as long as patient consent was obtained before surgery. Frozen-section or one-step nucleic-acid amplification were not performed.

### Study endpoints

Successful SLND was defined as the retrieval of at least one SLN with the respective technique. Concordance per procedure was defined as the proportion of procedures with at least one concordant SLN for both tracers divided by the procedures with at least one SLN detected with the radioisotope ((SPIO and radioisotope)/radioisotope). Reverse concordance per procedure was defined respectively ((SPIO and radioisotope)/SPIO). The number of SLN retrieved per technique was documented. Nodal and reverse concordance were calculated similarly. This study was registered in clinicaltrials.gov (NCT05985551) and undertaken to inform the design of the SENTINEO study (NCT05625698).

### Sample size, statistical analysis, and reporting

For SPIO administration before PST to be clinically meaningful, SPIO detection should be comparable to radioisotope-based detection and with high concordance that would be unaffected by the timing of administration. For that, a maximum absolute value of 0.3 was set as the tolerance margin for Spearman’s rho correlation coefficient and a maximum discordance of 8% in detection rates, presuming non-inferior detection rate for SPIO by 5%. The sample size satisfying both conditions was 114 patients. Sample size calculations were performed using G*Power version 3 (Dusseldorf University) and STATA version 16.

Categorical variables are summarized as *n* (%) with 95% confidence intervals. Paired comparisons were performed using McNemar’s test and non-paired comparisons were performed using Fisher’s test. Continuous variables are summarized as median (interquartile range (i.q.r.)) or median (range). Comparisons were made with the respective parametric or non-parametric test. Correlation of outcomes with the timing of SPIO administration was assessed using Kendall’s tau and Spearman’s rho. Multivariable analysis was performed if statistically significant differences were seen in the univariable analysis and regression models included the clinically relevant variables that were found to have significant interaction on univariable analysis. For these outcomes, standard and exponentiated B (expB) coefficients with 95% confidence intervals are reported for linear and logistic regression, respectively. Statistical analysis was performed using SPSS^®^ (IBM, Armonk, NY, USA; version 28) and Stata (StataCorp, College Station, TX, USA; version 17). Reported tests and *P* values are two-sided, unless stated otherwise. Continuity corrections were not performed. The manuscript was prepared and reported according to the STROBE statement^12^.

## Results

In total, 128 patients were eligible for this study. After PST completion, eight patients had a non-complete radiologic axillary response, six patients opted for axillary lymph node dissection, and one patient withdrew consent, leaving 113 patients for analysis (*Table 1*). Administration of SPIO was performed less than or equal to 1 week before surgery for 75 patients (66.4%) and greater than 1 week before surgery for 38 patients (33.6%; with 18.6% of patients receiving SPIO before the start of PST), at a median of 3 (range 0–248) days before surgery for the entire cohort.

**Table 1:** Study population characteristics

Age (years), median (i.q.r.)	56 (45–68)
BMI (kg/m^2^) median (i.q.r.)	25.1 (22.9–28.8)
Tumour size at baseline (mm), median (i.q.r.)	30 (22–42.5)
**T stage before PST**	
cT1	22 (19.6)
cT2	78 (69.7)
cT3	12 (10.7)
cT4	1 (0.9)
**N stage before PST**	
cN0	81 (71.1)
cN1	33 (28.9)
**Histology**	
IDC (NST)	103 (91.2)
ILC	7 (6.2)
Other (mucinous, medullar, metaplastic)	3 (2.7)
**Receptor status**	
HR+HER2−	41 (36.3)
HR+HER2+	17 (15.0)
HR−HER2+	17 (15.0)
HR−HER2−	38 (33.7)
**Type of PST**	
Chemotherapy ± targeted therapy	87 (77.7)
Endocrine therapy	25 (22.3)
**Duration of PST (days), median (i.q.r.)**	
Chemotherapy ± targeted therapy	145 (142–145)
Endocrine therapy	50 (45–91)

Values are *n* (%) unless otherwise indicated. i.q.r., interquartile range; PST, primary systemic therapy; IDC, invasive ductal cancer; NST, non-special type; ILC, invasive lobular cancer; HR, hormone receptor; HER2, human epidermal growth factor receptor 2.

At least one SLN was detected for 110 patients (97.3%) with SPIO and for 103 patients (91.2%) with radioisotope (difference 6.2%, 95% c.i. −0.8% to 13.2%; *P* = 0.057), whereas the combination of SPIO + radioisotope was successful for all patients (100%). Successful SPIO detection interacted negatively with higher BMI and administration on the day of surgery in the univariable analysis, but the effect was not retained in logistic regression, whereas radioisotope-based detection did not interact with any baseline factor. The addition of SPIO to radioisotope significantly increased the overall detection rate (difference 8.8%, 95% c.i. 2.4% to 15.0%; *P* < 0.001), but the addition of radioisotope to SPIO did not significantly improve overall detection (difference 2.7%, 95% c.i. −11.9% to 6.5%; *P* = 0.125). At least one SLN was concordant for SPIO and radioisotope in 100 of 113 procedures (88.5%, 95% c.i. 82.2% to 94.8%). The procedural concordance for radioisotope (‘magnetic and isotopic/isotopic’) was 97.1% (95% c.i. 93.8% to 100%) and the procedural concordance for SPIO (‘magnetic and isotopic/magnetic’) was 90.9% (95% c.i. 85.5% to 96.3%). Procedural concordance did not correlate with timing of SPIO injection (Kendall’s tau 0.027, 95% c.i. −0.098 to 0.151; *P* = 0.746).

Looking specifically into the successful identification of greater than or equal to two SLN (*Table 2*), SPIO were successful for 84.1% of patients and radioisotope was successful for 77.0% of patients (difference 7.1%, 95% c.i. −0.6% to 14.8%; *P* = 0.049). For both SPIO and radioisotope, only older age, higher BMI, and use of preoperative endocrine therapy interacted with probability for retrieval of less than two SLN. In logistic regression, none of these factors retained significance for radioisotope, but older age (Exp(B) = 0.922, 95% 0.871, 0.976; *P* = 0.005) and higher BMI (Exp(B) = 0.830, 95% 0.737, 0.935; *P* = 0.002) retained this effect for SPIO. Clinical axillary status at baseline (cN0 *versus* cN1) did not interact with the outcomes. The combination of SPIO + radioisotope detected greater than or equal to two SLN for 90.3% of patients, significantly different compared with SPIO only (difference 6.2%, 95% c.i. 0.9% to 11.5%; *P* = 0.008) or radioisotope only (difference 13.3%, 95% c.i. 6.1% to 20.3%; *P* < 0.001).

**Table 2:** Patients with different numbers of sentinel lymph nodes excised per technique

	Concordant*	SPIO only	Radioisotope only	SPIO + radioisotope combined
0	13 (11.5)	3 (2.7)	10 (8.8)	0 (0.0)
≥1	100 (88.5)	110 (97.3)	103 (91.2)	113 (100.0)
≥2	76 (67.3)	95 (84.1)	87 (77.0)	102 (90.3)
≥3	38 (33.6)	63 (55.8)	55 (48.7)	75 (66.4)

Values are *n* (%). *For concordant cases, 0 denotes a successful procedure, but no concordant sentinel lymph nodes. SPIO, superparamagnetic iron oxide nanoparticles.

Setting the threshold to greater than or equal to three SLN, SPIO were successful for 55.8% of patients and radioisotope was successful for 48.7% of patients (difference 7.1%, 95% c.i. −2.6% to 16.7%; *P* = 0.122). The combination of SPIO + radioisotope was successful for 66.4% of patients, significantly higher than for SPIO only (difference 10.6%, 95% c.i. 4.9% to 16.3%; *P* < 0.001) or radioisotope only (difference 17.7%, 95% c.i. 9.8% to 25.6;% *P* < 0.001).

A total of 356 SLN were identified with either SPIO or radioisotope. Out of these, 314 were detected by SPIO and 266 were detected by radioisotope; 226 SLN were concordant for SPIO and radioisotope. Thus, the nodal detection rate was 88.5% for SPIO and 75.0% for radioisotope (difference 13.5%, 95% c.i. 7.1% to 19.9%; *P* < 0.001). The median SLN yield was three (i.q.r. 2–3) for SPIO and two (i.q.r. 2–3) for radioisotope, resulting in a significant difference (*P* < 0.001). The median number of SLN for the combination of SPIO + radioisotope was three (i.q.r. 2–4), higher than for any single tracer (*P* < 0.001), whereas a median of two (1–3) SLN were concordant for SPIO and radioisotope. The nodal concordance was 85.0% (95% c.i. 80.1% to 89.0%) for radioisotope and the reverse concordance (SPIO) was 72.0% (95% c.i. 66.7% to 76.9%).

For cN+-to-ycN0 patients undergoing TAD (33 patients), the detection rate was 100% for SPIO (33 patients) and 82% for radioisotope (27 patients) (difference 18%, 95% c.i. 2% to 34%; *P* = 0.016). The index node was retrieved in all cases and was SPIO-positive in 31 (94%) and radioactive in 22 (67%) (difference 27%, 95% c.i. 7% to 48%; *P* = 0.007), an outcome that was not affected by age, BMI, type of PST, or time from SPIO and radioisotope injection to surgery. Overall, the median number of SLN identified using SPIO was higher than that identified using radioisotope (3 (i.q.r. 3–5) *versus* 2 (i.q.r. 2–3), respectively; *P* < 0.001). Specifically the TAD technique, compared with SLND, retrieved more SLN for SPIO (median of 3 (i.q.r. 3–5) *versus* 2 (i.q.r. 2–3), respectively; *P* < 0.001), but not for radioisotope (median of 2 (i.q.r. 2–3) for both; *P* = 0.875), whereas the number of concordant SLN did not differ (median of 2 (i.q.r. 2–3) *versus* 1 (i.q.r. 2–3) respectively; *P* = 0.273).

A median of one (i.q.r. 1–2) axillary metastasis was found in 19 patients (17%). For greater than or equal to one SLN, SPIO detection was 19 of 19 (100%) and radioisotope-based detection was 16 of 19 (84%) (difference 16%, 95% c.i. −0.6% to 32.2%; *P* = 0.083), for greater than or equal to two SLN, SPIO detection was 18 of 19 (95%) and radioisotope-based detection was 15 of 19 (79%) (difference 16%, 95% c.i. −0.6% to 32.2%; *P* = 0.083), and, for greater than or equal to three SLN, SPIO detection was 13 of 19 (68%) and radioisotope-based detection was nine of 19 (47%) (difference 21%, 95% c.i. 2.7% to 39.4%; *P* = 0.046). In this subgroup of ypN+ patients, SPIO detected more SLN than radioisotope (median of 3 (i.q.r. 2–4) *versus* 2 (i.q.r. 2–3) respectively; *P* = 0.010) and more metastatic SLN than radioisotope (median of 1 (i.q.r 1–2) *versus* 1 (i.q.r. 0–1) respectively; *P* = 0.005). From those patients that underwent completion axillary lymph node dissection, additional metastatic nodes were found in one patient (4%).

Time from SPIO administration to surgery did not affect the number of SPIO SLN (Spearman’s rho 0.053, 95% c.i. −0.138 to 0.241; *P* = 0.575) or nodal concordance (Spearman’s rho −0.022, 95% c.i. −0.220 to 0.177; *P* = 0.821). In univariable analysis, the number of SPIO SLN interacted with patient age, BMI, and positive clinical nodal status at baseline (*Table 3*). Linear regression showed a persisting negative effect between number of SPIO SLN and BMI, and a positive interaction between number of SPIO SLN and positive axillary status at baseline. The number of radioisotope SLN interacted with BMI only, an effect retained on multivariable analysis, with higher BMI resulting in retrieval of less radioisotope SLN.

**Table 3:** Factors affecting numbers of sentinel lymph nodes identified per technique

	Univariable analysis		Multivariable analysis	
	Spearman’s rho (95% c.i.)	*P*	Coefficient b (95% c.i.)	*P*
**SPIO**				
Age	−0.022 (−0.447,−0.096)	0.003	−0.018 (−0.036,0.000)	0.055
BMI	−0.334 (−0.498,−0.147)	<0.001	−0.090 (−0.141,−0.038)	<0.001
cN stage	0.245 (0.056,0.416)	0.009	0.758 (0.134,1.383)	0.018
**Radioisotope**				
Age	−0.171 (−0.350,0.019)	0.069	−0.009 (−0.026,0.009)	0.318
BMI	−0.334 (−0.497,−0.147)	<0.001	−0.051 (−0.101,−0.002)	0.041
cN stage	−0.013 (−0.204,0.178)	0.890	−0.031 (−0.629,0.568)	0.919

Multivariable analysis is linear regression for the factors with significant correlation. SPIO, superparamagnetic iron oxide nanoparticles; cN stage, clinical node stage at presentation.

All patients who received SPIO before PST (21 patients; median of 135 (i.q.r. 120–140) days) had successful magnetic SLND, whereas radioisotope was successful for 17 of 21 (difference 19%, 95% c.i. 2.3% to 35.8%; *P* = 0.046). For these 21 patients, a median of 3 (i.q.r. 2–3) SPIO SLN were retrieved and the median number of concordant SLN was two (i.q.r. 1–3). The median magnetic count of the retrieved SLN for these patients was lower compared with that for the rest of the cohort (1430 *versus* 2523 respectively; *P* = 0.002), but there was no difference in the median magnetic count of the first SLN detected (4100 *versus* 3873 respectively; *P* = 0.567).

One patient who received SPIO 2 days before surgery presented with mild skin staining that disappeared at 4 weeks after surgery. No other adverse events were reported.

## Discussion

For this well-defined cohort of patients undergoing SLND or TAD after PST, it was shown that SPIO performed comparably to radioisotope, but detected more SLN and had a higher rate of detection of metastatic SLN. Moreover, administration before PST did not affect concordance with radioisotope, meaning that SPIO provide the possibility of mapping the axilla before PST.

Axillary mapping after PST has been established as the standard of care, as the feasibility and accuracy of the procedure have been demonstrated for both cN0 and cN+-to-ycN0 patients in larger studies^4,6,13,14^. Initial concerns have largely been abandoned, as it has been shown that ypN is a stronger prognosticator than cN^15,16^. In two meta-analyses, conducted in 2009 and 2022, the pooled SLND rate was, however, 90.9% and 90.6% respectively, with significant heterogeneity (*I*^2^ = 89%)^17,18^. While no difference in this outcome was reported with regard to baseline axillary status, previous literature suggests that radioisotope ± blue dye outperforms blue dye alone^4,5,13,14,19^. In the present study, SPIO detection was very high and was not affected by baseline axillary status, a finding consistent with previous reports^20^, whereas radioisotope-based detection was comparable to the available literature^17^. The negative interaction between high BMI and SPIO detection does not seem to be tracer-specific, as high BMI has been identified as a challenge for other tracers as well^18,21^. Additionally, the detection rate of SPIO was comparable to that of the combination of SPIO + radioisotope, suggesting a potential advantage over radioisotope in its use as sole tracer. This is an advantage compared with other isotope-free tracers, such as blue dye, which performs worse than radioisotope^4,5,19^, or indocyanine green, which performs comparably to radioisotope, but without any benefit compared with the combination of indocyanine green + radioisotope^22^.

With regard to SLND after PST, a concern beyond detection is accuracy, especially for cN+-to-ycN0 patients, for whom false negative rates under 10% have repeatedly been associated with the retrieval of greater than or equal to three SLN^4,6,14^. The introduction of TAD has facilitated this and decreased false negative rates even more^5–7^, but surgeons often encounter the phenomenon of retrieving less than three SLN in these patients. Institutional reports suggest that three SLN may not be an absolute cut-off, but it is unclear whether higher axillary recurrences were observed with the retrieval of only one SLN *versus* two SLN^23^. However, adequate nodal yield should not aim at the prevention of axillary recurrence, but accurate staging. This is important, as residual disease may affect treatment decisions^24–27^ and prompt completion axillary dissection, until the role of radiotherapy has been elucidated^28^. The use of SPIO resulted in high detection rates, retrieving a median of three SLN, regardless of baseline cN. The clipped lymph node was an SLN for SPIO in 94% of cases, whereas it was an SLN for radioisotope in only 67% of cases, the latter being consistent with previous studies^29^. Interestingly, despite the fact that the combination of SPIO + radioisotope had a higher probability of retrieving more SLN, this was not significant for patients with malignant SLN. This observation is important, as it may hint at possibilities for more accurate axillary staging. Such a finding could be explained by the fact that SPIO is taken up by tissue macrophages in the lymph node and that SPIO maps SLN before the fibrotic effect of PST, the latter contributing to SLND failure^9^. This should be viewed as hypothesis generating and should be tested in a dedicated trial.

A novel finding of this study is that axillary mapping before PST is feasible and does not affect procedural accuracy. Indeed, no association between timing of SPIO administration and concordance between radioisotope and SPIO could be found, thus satisfying the primary outcome of this study. Moreover, the detection rate and the nodal yield for the patients receiving SPIO before PST were comparable to those for the rest of the cohort. The median magnetic count was lower for ‘all SLN’, but not for the ‘first SLN’, and the values allowed for easy detection. These data not only corroborate previous reports and meta-analyses regarding SPIO as a tracer for SLN after PST^1,20^ but suggest that the concept of delayed SLND through a wide time frame between SPIO administration and SLND, introduced in the SentiNot study^2,3^, can be applicable in the setting of PST, facilitating logistics and potentially enhancing axillary mapping.

This study has certain limitations. It is a feasibility study, primarily assessing the interplay between the timing of SPIO administration and concordance between SPIO and radioisotope, as the latter was administered according to clinical routine. The outcomes are interesting and clearly suggest that SPIO can be used for SLND after PST, but the implementation of a prolonged time frame needs to be tested in a dedicated trial. Moreover, SPIO administration before PST precludes the possibility of MRI monitoring, currently a popular strategy^30^. This, reassuringly, does not constitute a major limitation, as the literature suggests that MRI is not superior to ultrasonography when assessing the response in the breast^31,32^ or the axilla^33^. Moreover, a recent meta-analysis suggests that contrast-enhanced mammography, a modality that does not interfere with SPIO, seems to yield comparable diagnostic accuracy to MRI during PST^34^. Thus, the potential of axillary mapping with SPIO before PST should be explored, especially in light of the findings of the present study. Finally, this is a single-centre study, from an institute with extensive experience with the magnetic technique, suggesting that the results should be externally validated. Currently, the SENTINEO pilot study^35^ is accruing data and a multicentre trial is being planned.

## Data Availability

An application for data sharing can be made available upon reasonable request following contact with the corresponding author.
